# Retraction Note: Transplantation of Mouse Induced Pluripotent Stem Cell-Derived Podocytes in a Mouse Model of Membranous Nephropathy Attenuates Proteinuria

**DOI:** 10.1038/s41598-021-93174-z

**Published:** 2021-07-05

**Authors:** Amin Ahmadi, Reza Moghadasali, Vahid Ezzatizadeh, Zeinab Taghizadeh, Seyed Mahdi Nassiri, Mohammad Hassan Asghari-Vostikolaee, Mehdi Alikhani, Fatemeh Hadi, Reza Rahbarghazi, Reza Salman Yazdi, Hossein Baharvand, Nasser Aghdami

**Affiliations:** 1 Department of Stem Cells and Developmental Biology, Cell Science Research Center, Royan Institute for Stem Cell Biology and Technology, ACECR, Tehran, Iran; 2 Medical Genetics Department, Medical Laboratory Center, Royesh Medical Group, Tehran, Iran; 3 Department of Clinical Pathology, Faculty of Veterinary Medicine, University of Tehran, Tehran, Iran; 4 Animal Core Facility, Reproductive Biomedicine Research Center, Royan Institute for Biotechnology, ACECR, Tehran, Iran; 5 Department of Brain and Cognitive Sciences, Cell Science Research Center, Royan Institute for Stem Cell Biology and Technology, ACECR, Tehran, Iran; 6 Stem Cell Research Center, Tabriz University of Medical Sciences, Tabriz, Iran; 7 Department of Andrology, Reproductive Biomedicine Research Center, Royan Institute for Reproductive Biomedicine, ACECR, Tehran, Iran; 8 Department of Developmental Biology, University of Science and Culture, Tehran, Iran; 9 Department of Regenerative Medicine, Cell Science Research Center, Royan Institute for Stem Cell Biology and Technology, ACECR, Tehran, Iran

Retraction of: *Scientific Reports* 10.1038/s41598-019-51770-0, published online 29 October 2019

 The authors have retracted this Article.

After publication, the authors reported several irregularities in the histopathology images presented, specifically:

- Figure 3A: The H&E/PI panel looks identical to the PI/H&E (day 20) panel in Figure 4.
- Figure 3A: The MT/NT panel looks identical to the PI/MT (day 60) panel in Figure 4.
- Figure 4: The APN + PBS/PAS (day 50) panel was edited to bring the glomeruli closer together.

Amin Ahmadi, Vahid Ezzatizadeh, Mehdi Alikhani, Fatemeh Hadi, and Hossein Baharvand agree to this retraction. Seyed Mahdi Nassiri does not agree with this retraction. Reza Moghadasali, Zeinab Taghizadeh, Mohammad Hassan Asghari-Vostikolaee, Reza Rahbarghazi, Reza Salman Yazdi, and Nasser Aghdami did not respond to correspondence relating to this retraction.

